# What is the optimal input information for deep learning-based pre-treatment error identification in radiotherapy?

**DOI:** 10.1016/j.phro.2022.08.007

**Published:** 2022-08-27

**Authors:** Cecile J.A. Wolfs, Frank Verhaegen

**Affiliations:** Department of Radiation Oncology (Maastro), GROW – School for Oncology and Reproduction, Maastricht University Medical Centre+, Maastricht, the Netherlands

**Keywords:** Deep learning, Artificial intelligence, Quality assurance, Error detection, Pre-treatment verification, Gamma analysis

## Abstract

**Background and purpose:**

Deep learning (DL) provides high sensitivity for detecting and identifying errors in pre-treatment radiotherapy quality assurance (QA). This work’s objective was to systematically evaluate the impact of different dose comparison and image preprocessing methods on DL model performance for error identification in pre-treatment QA.

**Materials and methods:**

For 53 volumetric modulated arc therapy (VMAT) and 69 stereotactic body radiotherapy (SBRT) treatment plans of lung cancer patients, mechanical errors were simulated (MLC leaf positions, monitor unit scaling, collimator rotation). Two classification levels were assessed: error type (Level 1) and error magnitude (Level 2). Portal dose images with and without errors were compared using standard (gamma analysis), simple (absolute/relative dose difference, ratio) and alternative (distance-to-agreement, structural similarity index, gradient) dose comparison methods. For preprocessing, different normalization methods (min/max and mean/standard deviation) and image resolutions (32 × 32, 64 × 64 and 128 × 128) were evaluated. All possible combinations of classification level, dose comparison, normalization method and image size resulted in 144 input datasets for DL networks for error identification.

**Results:**

Average accuracy was highest for simple dose comparison methods (Level 1: 97.7%, Level 2: 78.1%) while alternative methods scored lowest (Level 1: 91.6%, Level 2: 71.2%). Mean/stdev normalization particularly improved Level 2 classification. Higher image resolution improved error identification, although for SBRT lower image resolution was also sufficient.

**Conclusions:**

The choice of dose comparison method has the largest impact on error identification for pre-treatment QA using DL, compared to image preprocessing. Model performance can improve by using simple dose comparison methods, mean/stdev normalization and high image resolution.

## Highlights

• The choice of dose comparison method impacts deep learning error identification accuracy most.
• Simple dose comparison methods are more beneficial than gamma analysis and alternative methods.
• Mean/standard deviation normalization and high image resolution improve error identification.

## 1 Introduction

In pre-treatment patient specific quality assurance (QA), the radiotherapy treatment plan is delivered to a measurement device before the actual patient is treated, to evaluate deliverability of the plan. This is, for instance, done using electronic portal imaging device (EPID) dosimetry. Gamma analysis between predicted and acquired two dimensional (2D) EPID dose distributions, with standard dose difference (DD) and distance-to-agreement (DTA) criteria, and fixed thresholds on gamma pass rates are commonly used for dose comparison and error detection [1], [2]. However, this approach has inherent limitations. Gamma pass rates from 2D dose measurements have been shown to have limited correlation to clinically relevant differences in dose-volume histogram metrics based on the three dimensional (3D) patient dose [3], [4], [5], [6], and the wealth of 2D or 3D measurement data is reduced to a few metrics. Furthermore, different treatment modalities may require different gamma criteria and pass/fail rate thresholds, while in clinical practice often the same criteria are used for all treatments [7], [8], [9]. For instance, for stereotactic body radiotherapy (SBRT) treatments with small radiation fields and high doses, less strict dose difference (DD) but stricter distance-to-agreement (DTA) criteria may be warranted [10].

As in many other parts of the radiotherapy workflow, artificial intelligence (AI) has been utilized in pre-treatment QA for improving error detection sensitivity and efficiency [11], [12]. Several studies have focused on not only detecting errors based on gamma pass rates, but also on identifying their causes, using deep learning (DL) algorithms that can take full dose comparison images as input [13], [14], [15], [16], [17]. This DL approach does not have the limitations of traditional error detection systems, as it is not necessary to reduce 2D or 3D dose measurements to a few metrics, which allows for extracting more information (e.g., the root cause of the error) than was traditionally possible. These studies show promising results, with DL models providing high sensitivity for detecting and identifying errors and additional information on error causes that cannot be obtained with traditional gamma pass/fail rates.

While gamma analysis is the traditional dose comparison method of choice in clinical practice, other comparison methods (e.g., DD maps) may provide more information for DL models for error identification. Although the comparison images resulting from different comparison methods can appear too noisy for human interpretation, DL models may be able to utilize this additional information. This way, error detection and identification using DL can potentially be improved further. Another factor influencing DL model performance is image preprocessing, which is an inherent step in any DL method [18]. Different image resolutions or normalization methods could provide better DL performance. Furthermore, the optimal combination of dose comparison method, image resolution and image normalization can also differ for different treatment modalities (e.g., regular volumetric modulated arc therapy (VMAT) vs. SBRT).

The objective of this work was to systematically evaluate the impact of different dose comparison and image preprocessing methods on the performance of a DL model for error identification in pre-treatment QA. To this end, a large database was created by simulating errors and pre-treatment dose distributions. This database was used to systematically test combinations of dose comparison and image preprocessing methods, to determine the combination that leads to the highest DL model performance for both regular VMAT and SBRT treatment plans of lung cancer patients.

## 2 Materials and methods

Two 2D dose distribution datasets were created, based on 53 regular VMAT and 69 SBRT treatment plans of 46 and 63 lung cancer patients, respectively. The fractionation schemes of the regular VMAT plans were 24 × 2.75, 30 × 1.8 or 33 × 2 Gy, and those of the SBRT plans were 3 × 15 or 4 × 12 Gy. Each treatment plan consisted of two arcs, with the exception of two SBRT plans that contained three arcs. The average amount of monitor units (MU) per arc (±standard deviation) was 333 ± 85 for the regular VMAT plans and 1746 ± 251 for the SBRT plans.

Mechanical errors were simulated by changing parameters in the treatment plans. The simulated errors and their magnitudes are listed in Table 1. Collimator rotation was simulated by changing the angle of the collimator. To simulate multileaf collimator (MLC) errors, the leaf positions were adjusted. In the systematic MLC error case, one or both leaf banks were shifted as a whole, while in the random MLC error case, each leaf position was adjusted individually. Monitor unit (MU) errors were simulated by scaling the MU values by a certain percentage. For each error, 20 simulations per treatment plan for different error magnitudes were performed. In the cases of collimator rotation, systematic MLC and systematic MU errors, the same error magnitude was applied to all segments in a treatment arc, while for the random MLC and MU errors the error magnitude differed per segment and was averaged afterwards to obtain one overall value.

**Table 1:** Overview of the simulated mechanical errors and their magnitudes. MLC: multileaf collimator; MU: monitor unit.

Error type	Error magnitude (excluding 0) [step size]	Error magnitude threshold (absolute value)
Collimator rotation	−2 to +2° [0.2]	1°
MLC systematic	−2 to +2 mm [0.2]	1 mm
MLC random	−2 to +2 mm [0.2]	0.5 mm
MU systematic	−10 to +10% [1]	5%
MU random	−10 to +10% [1]	3%

Two classification levels were assessed, with Level 1 corresponding to classification of the error type and Level 2 to classification of the error magnitude. For Level 2 classification, the thresholds listed in Table 1 separated the error magnitudes in two classes, that can be interpreted as relevant and irrelevant errors. These thresholds were determined for the purposes of this study. For use in clinical practice, they should be optimized and their clinical relevance should be evaluated. As errors were simulated per segment of the treatment arcs, random errors may average out when the dose per segment is summed up into an integrated 2D dose distribution [19], [20]. However, while errors may average out in the dose measurement, that does not necessarily mean that their clinical consequences also average out. Therefore, to prevent missing potentially clinically relevant errors, stricter thresholds were chosen for the random errors.

For all treatment plans, 2D time-integrated portal dose images were predicted using an in-house developed 2D pre-treatment dose prediction model [21]. This model was implemented in Matlab (v9.7, Mathworks, Natick, MA, USA), and predicts the dose in a plane in a virtual homogenous phantom in full scatter conditions. It was fitted for the TrueBeam and TrueBeam STx (Varian Medical Systems, Palo Alto, CA, USA). The dose images were simulated at a source-detector distance of 100 cm.

**Fig. 1:**
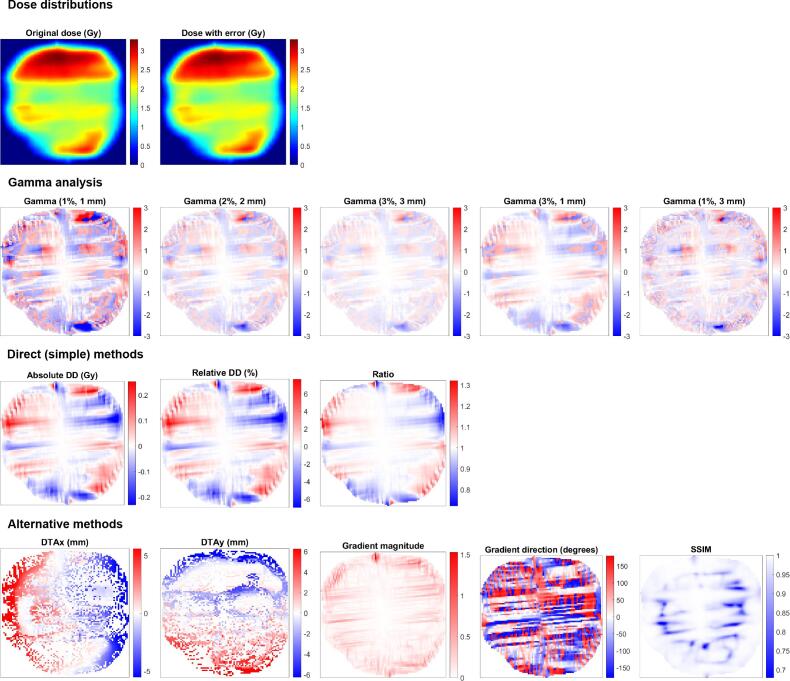
Examples of dose distributions and corresponding dose comparison images. For the dose with error, a 2° collimator rotation was simulated. For all dose comparison images, the white color represents areas with no error, while red and blue represent areas of error with positive and negative values, respectively. The interpretation of positive and negative values depends on the dose comparison method. E.g., for gamma analysis and dose difference maps, red represents overdosage, while for the DTAx it represents a distance in the positive direction (i.e., to the right). The colormaps are for visualization purposes only; the underlying pixel values in the images are used as input for the preprocessing workflow and subsequently the DL network. DD: dose difference; DTA: distance-to-agreement; SSIM: structural similarity index.

The dose based on a plan with a simulated error was then compared to the dose based on the plan without error, using various dose comparison methods. In total, seven different dose comparison methods were applied (Table 2). These dose comparison methods were chosen because they are commonly used in radiotherapy (i.e., gamma analysis), because of their simplicity (i.e., ratio and DD maps) or because they are not commonly used for this purpose but could provide beneficial information for a DL network (i.e., DTA maps, DD and DTA separately, structural similarity index (SSIM) [22] and the gradient method). An example comparison image of each of the dose comparison methods is provided in Fig. 1. All dose comparisons were performed in Matlab using either standard available functions or in-house developed software [23].

**Table 2:** Overview of the systematically evaluated input factors: dose comparison method, image normalization method and image resolution. In the gamma analysis, global dose differences with respect to the maximum dose in the reference dose distribution were considered. DD: dose difference; DTA: distance-to-agreement; SSIM: structural similarity index; stdev: standard deviation.

**Dose comparison method**	Gamma analysis	(1%, 1 mm)
		(2%, 2 mm)
		(3%, 3 mm)
		(3%, 1 mm)
		(1%, 3 mm)
	Ratio	Dose with error divided by dose without error
	DD	Absolute: DD per pixel
		Relative: DD per pixel divided by the maximum dose
	DTA	Distance to pixel with corresponding dose value in x (row) and y (column) directions
	DTA/DD	Combination of relative DD, DTA in x direction and DTA in y direction (i.e., all components of gamma analysis separately)
	SSIM	Similarity between two images, based on • Luminance: intensity of the recorded object (i.e., the image’s pixel values) • Contrast: difference/variation in luminance • Structure: correlation of the luminance of two images A mathematical description of the SSIM is provided by Peng et al. [22]
	Gradient	Magnitude and direction of gradients in DD maps based on the Sobel gradient operator [31], [32]

**Image normalization**	Min/max	• Calculate mean and standard deviation of dataset • Minimum/maximum values = mean ± 2 · standard deviation • Clip pixel values to minimum and maximum values • Calculate $\frac{\text{pixel value}-\min\;\text{value}}{\max\;\text{value}-\min\;\text{value}}$ for each image
	Mean/stdev	• Calculate mean and standard deviation of dataset • Calculate $\frac{\text{pixel value}-\text{mean}}{\text{standard deviation}}$ for each image

**Image resolution**	32 × 32	
	64 × 64	
	128 × 128

While most dose comparison methods resulted in single-channel images as input for the DL network, the DTA, DD/DTA and gradient methods resulted in multi-channel images. The DTA was calculated in the x and y directions separately, and was input as a two-channel image into the DL network. The addition of the relative DD map led to a three-channel input for the DD/DTA method. For the gradient method, the magnitude and direction of the gradients are calculated separately, also leading to a two-channel input for the DL network.

Image preprocessing consisted of cropping the dose comparison images to the radiation field by applying a 10% low dose threshold, normalizing the images and resizing to a square image size. Image normalization is a standard step in preprocessing images for DL, to increase the efficiency of model training. Normalization was applied to the dose comparison images and did not affect the dose distributions directly. Two types of normalization were evaluated: 1) clipping the pixel values to a minimum and maximum value and normalizing them to the range [0,1] and 2) subtracting the mean value from the pixel values and dividing by the standard deviation. A detailed explanation of these methods is provided in Table 2. For the square image size, three options were chosen, ranging from relatively small (32 × 32) to large (128 × 128). As cropping of the images before resizing to a square image size was based on a 10% low dose threshold, the size of the cropped images and consequently the pixel size in the square images depended on the radiation field size and could differ between different treatment plans and modalities. An overview of the average field, image and pixel sizes of the datasets is provided in Table 3. Making all possible combinations of classification level, dose comparison method, normalization method and image size led to 144 input datasets of 10.440 images each for the regular VMAT plans and 144 input datasets of 13.720 images each for the SBRT plans. These were split in training (70%), validation (10%) and test (20%) sets.

**Table 3:** Overview of the radiation field, cropped image and pixel sizes for the different treatment plans. All reported values are the average length × width over all treatment arcs. The pixel size of the original dose images is 0.8 × 0.8 mm.

	VMAT	SBRT
Field size (cm)	9.5 × 8.7	4.9 × 4.7
Number of pixels after cropping based on 10% low dose threshold	118 × 121	57 × 68
Pixel size after resizing to 32 × 32 pixels (mm)	2.9 × 3.0	1.4 × 1.7
Pixel size after resizing to 64 × 64 pixels (mm)	1.4 × 1.5	0.7 × 0.8
Pixel size after resizing to 128 × 128 pixels (mm)	0.7 × 0.7	0.3 × 0.4

A DL network architecture consisting of multiple blocks of two convolutional layers and a max pooling layer, followed by dense layers was implemented in Keras/Tensorflow [24]. The exact network architecture and hyperparameters (Supplementary Material A) were optimized for each input dataset using Bayesian optimization through the hyperparameter optimization framework Optuna [25]. The DL networks were trained on a 12 GB Titan Xp GPU (NVIDIA, Santa Clara, CA, USA). Early stopping based on validation loss was applied to prevent overfitting, and pruning to limit unnecessary exploration of unpromising hyperparameter configurations. Model performance was evaluated by calculating the accuracy, i.e., the percentage of images classified in the correct class. As the dataset was well balanced with respect to the number of images in each class, no other evaluation metric was considered. Training times were recorded to evaluate training speed.

## 3 Results

**Fig. 2:**
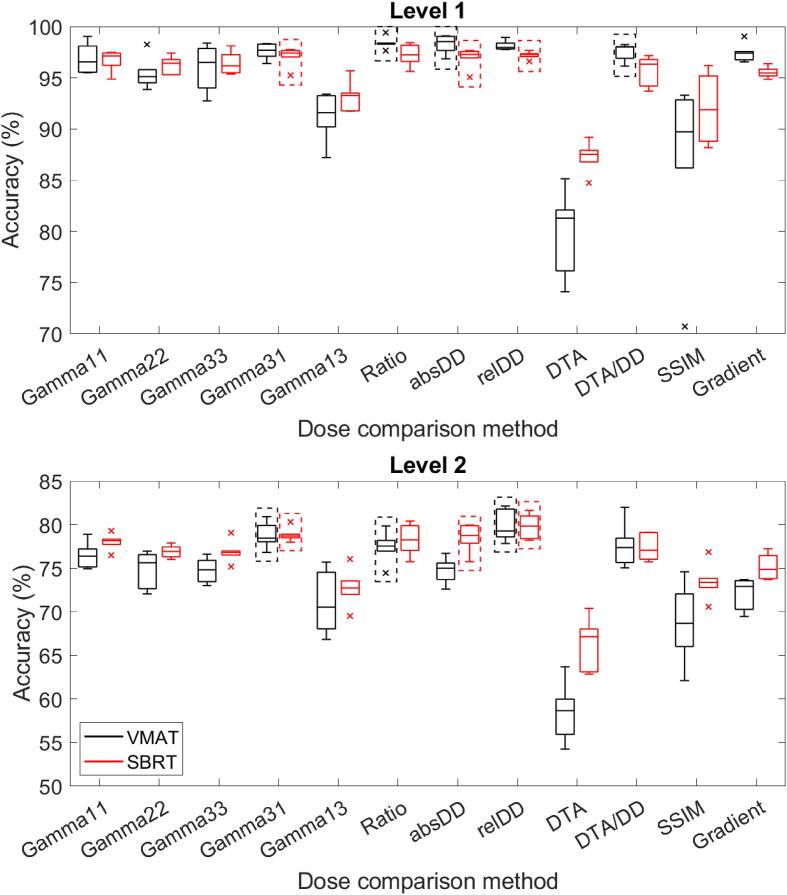
Deep learning network performance on the test dataset for different dose comparison methods. Level 1 corresponds to classification of the error type, Level 2 to classification of the error magnitude. The dashed boxes indicate the three methods with the highest median performance. GammaXY: (X%, Y mm) gamma map, relDD: relative dose difference, absDD: absolute dose difference, DTA: distance-to-agreement, SSIM: structural similarity index.

Fig. 2 shows that using relatively simple dose comparison methods such as ratio analysis (median accuracy Level 1: 98.4%/97.2%, Level 2: 77.6%/78.3% for VMAT/SBRT) or relative DD (Level 1: 97.8%/97.3%, Level 2: 79.3%/79.8%) provided highest DL model performance, although gamma analysis with strict criteria (particularly in the DTA; (3%, 1 mm) Level 1: 97.7%/97.4%, Level 2: 78.5%/78.7%) also performed well. Gamma analysis with strict relative DD but less strict DTA (i.e., (1%, 3 mm); Level 1: 91.6%/93.3%, Level 2: 70.6%/72.7%), DTA alone (Level 1: 81.3%/87.5%, Level 2: 58.7%/67.2%) and SSIM (Level 1: 89.7%/91.9%, Level 2: 68.7%/73.4%) did not perform well. The accuracy of the DL networks trained with these images as input was substantially, and for some cases even significantly (Supplementary Material B), lower than that of the other methods. The performance of the DL networks with the clinically commonly used gamma analysis with (3%, 3 mm) or (2%, 2 mm) criteria was close to the average over all dose comparison methods (Level 1: 94.5%/95.1%, Level 2: 73.7%/76.0%). The same trends are seen for Level 1 versus Level 2 classification, as well as for VMAT versus SBRT plans. As a result of the SBRT dataset being larger than the regular VMAT dataset, the SBRT results generally show smaller variance than the regular VMAT results.

**Fig. 3:**
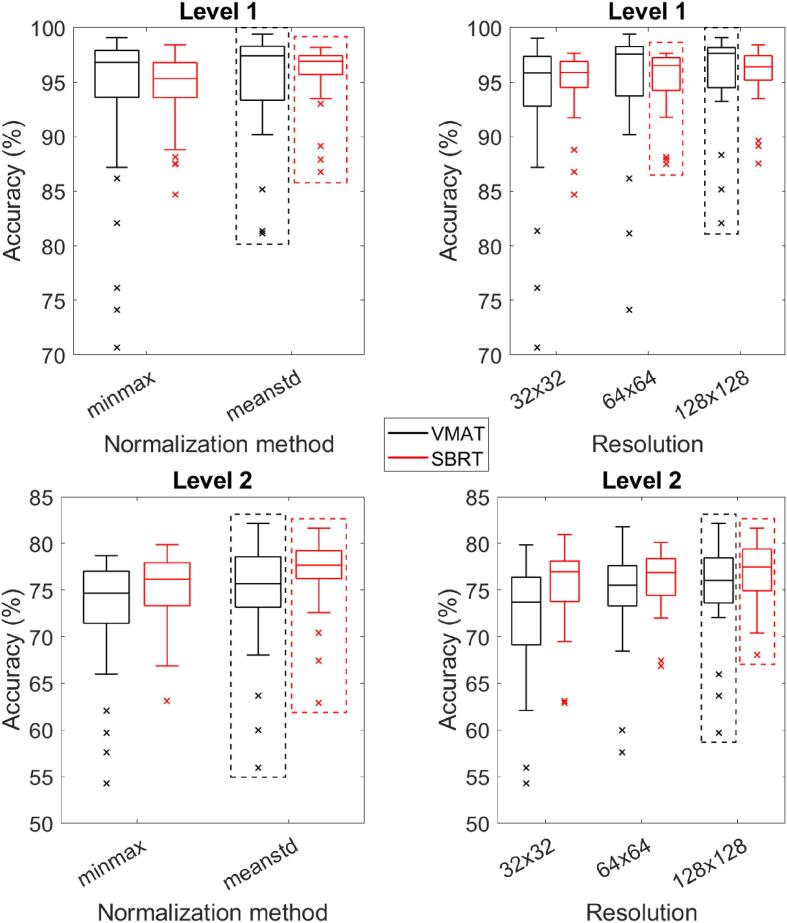
Deep learning network performance on the test dataset for different image normalization methods and image resolutions. Level 1 corresponds to classification of the error type, Level 2 to classification of the error magnitude. The dashed boxes indicate the normalization method/resolution with the highest median performance.

Regarding image normalization (Fig. 3), when using the mean/stdev method, DL performance was higher compared to using the min/max method. The difference between the two methods was slightly larger for SBRT plans than for regular VMAT plans, especially for Level 1 classification. Fig. 3 also demonstrates that median performance of the DL networks increased with higher image resolution. This preprocessing step had a larger influence on DL performance for regular VMAT plans than for SBRT plans, although none of the differences were statistically significant (Supplementary Material B). The average training times ± 1 standard deviation for 32 × 32, 64 × 64 and 128 × 128 were 87.7 ± 80.3, 151.6 ± 93.0 and 403.0 ± 193.2 s, respectively, on a 12 GB Titan Xp GPU (NVIDIA).

## 4 Discussion

A systematic evaluation of various types of input data of a DL network for pre-treatment error identification showed that the choice of dose comparison method had the largest influence on DL model performance. Using simple, direct dose comparison methods led to the highest DL model performance, with an increase in average accuracy of approximately 2 percentage points for Level 1 and up to 5 percentage points for Level 2, compared to standard (3%, 3 mm) gamma analysis. While these simple dose comparison methods lead to dose comparison images that may contain too many details for human interpreters and are therefore not used in clinical practice, they are beneficial for DL networks. Following this reasoning, it may also be beneficial to decrease the low dose threshold that was used to crop the dose comparison images, to determine if including larger low dose regions may further improve DL performance for error detection and identification.

Generally, it is not beneficial for DL performance to use the DTA, SSIM or gradient method, i.e., methods that are not commonly used for dose comparisons. Even though these highlight different information than clinically used methods (Fig. 1), they are not informative enough for DL networks to learn to identify which error occurred, with differences in accuracy up to −16 percentage points compared to (3%, 3 mm) gamma analysis. It could be beneficial to combine these methods with better scoring ones by using multiple image channels as input. However, from Fig. 2 it can be derived that combining the low scoring DTA method with a relative DD map (DTA/DD method) does increase DL performance, but not to the level of relative DD alone.

Besides providing highest DL model performance, using simple dose comparison methods also has practical benefits. In contrast to gamma analysis, there are no parameters such as dose or distance criteria associated with these dose comparison methods. While for gamma analysis a new DL model would need to be trained for each combination of DD and DTA criteria, this is not necessary for direct dose comparisons, making them more robust and flexible. Furthermore, implementing gamma analysis can be computationally expensive and challenging [26], while DD and ratio maps are easy and fast to compute.

The influence of image preprocessing methods on DL performance is small compared to the influence of the dose comparison method. Even though the mean/stdev method provides consistently better results than the min/max method, only for SBRT plans this difference is statistically significant (Supplementary Material B). Higher image resolution improves error identification, as more details of the dose comparison images are preserved. For SBRT plans, this effect is less pronounced. This is likely because the radiation fields in SBRT plans are smaller than in regular VMAT plans (Table 3), which leads to smaller image sizes already containing sufficient information for the DL network. Comparing average pixel sizes for the different treatment modalities (Table 3) to the original pixel size confirms that for regular VMAT, the average pixel size of the 128 × 128 images is closest to the original pixel size, while for SBRT this holds true for the 64 × 64 images. Hence, for SBRT the 64 × 64 images already contain all information that was in the original images, while for regular VMAT lower image resolution may blur some details. For SBRT, resizing to 128 × 128 will include extrapolation, which does not seem to hamper DL performance, but may introduce artifacts in the images. It should be noted that for training DL networks with higher image resolution more computational resources and longer training times are needed. While this is not a major issue for the 2D images used in this work, it may be for 2D images per timepoint or for 3D reconstructed dose volumes.

A limitation of this work is that all dose distributions were simulated, which means that no noise or other sources of delivery error (e.g., mechanical sag of the gantry and EPID during rotation of the linac) are present in the input data. In practice, measured dose distributions will contain measurement noise and uncertainty, which will propagate into the dose comparison images [27], [28]. This can potentially decrease DL model performance, as it will be more difficult to distinguish between noise and real errors. Gamma analysis might provide better DL performance in the presence of noise and measurement uncertainty than simple dose comparison methods, as gamma analysis can smooth out some of the noise. While small DTA provided best performance in this work, larger DTA may be needed to compensate for mechanical linac and EPID positioning deviations during delivery. However, this needs to be confirmed in future research. Furthermore, the thresholds distinguishing relevant from irrelevant errors in the Level 2 classification were chosen as a realistic example for this study, but should be optimized for use in clinical practice. Moreover, for dynamic treatments, the use of time-resolved instead of time-integrated verification could further improve error identification [29], [30]. However, this poses challenges for DL due to the vast increase in dataset size and computational resources needed to process this data.

To conclude, the choice of dose comparison method has the largest impact on error identification for pre-treatment QA using DL, compared to image preprocessing. Model performance can improve by using simple dose comparison methods such as relative DD or ratio maps, by applying mean/stdev normalization and by using high image resolution (128 × 128), although for SBRT treatment plans lower image resolution (64 × 64) is also sufficient.

## Declaration of Competing Interest

The authors declare that they have no known competing financial interests or personal relationships that could have appeared to influence the work reported in this paper.
