# Supplementary material for: What is the optimal input information for deep learning-based pre-treatment error identification in radiotherapy?
Source: Phys Imaging Radiat Oncol. 2022 Aug 27;24:14–20. doi: 10.1016/j.phro.2022.08.007 (PMC9465434; doi:10.1016/j.phro.2022.08.007)
Supplement: Supplementary Data 1 [file mmc1.pdf]

Supplementary Material A: Hyperparameter optimization

Table A1 shows the hyperparameters that were optimized for each input dataset. Using Optuna [1], 100 hyperparameter configurations were tested per dataset, where the first 25 were initialized randomly and the remaining were chosen using the results of the first 25 configurations and a Bayesian optimization process. The configuration with the highest validation accuracy was chosen as the final model.

Table A1: Overview of the optimized hyperparameters, their possible values and additional constraints to ensure proper hyperparameter configurations.

Hyperparameter	Possible values	Additional constraints
# convolutional blocks	[2, 3, 4, 5, 6]	Each convolutional block consists of two convolutional layers and a max pooling layer. Consequently, for image size 32x32, the maximum # convolutional blocks is 4, for 64x64 the maximum is 5 and for 128x128 the maximum is 6.
# filters in first convolutional layer	[8, 16, 32]	The two convolutional layers in each convolutional block have the same # filters.
# fully connected layers	[1, 2]	
# nodes in first fully connected layer	[64, 128, 512, 1024]	
Ratio between # nodes in consecutive dense layers	[2, 4, 8]	
Learning rate	[10^{-5} – 10^{-2}]	Continuous range, sampled in the logarithmic domain.
Dropout probability	[0 – 0.5]	Discrete range, with step size 0.05.
Batch size	[32, 64, 128, 256]	

[1] Akiba T, Sano S, Yanase T, Ohta T, Koyama M. Optuna: A Next-generation Hyperparameter Optimization Framework. Proceedings of the 25th ACM SIGKDD International Conference on Knowledge Discovery & Data Mining. Anchorage, AK, USA: Association for Computing Machinery; 2019. p. 2623–31.

Supplementary Material B: Statistical comparisons

To evaluate whether observed differences between dose comparison methods, image normalization methods and image resolutions were statistically relevant, pairwise comparisons using Kruskal-Wallis tests with Bonferroni correction for multiple testing were performed. The non-parametric Kruskal-Wallis test was chosen, as the data in each group was not always normally distributed (as confirmed by Shapiro-Wilk tests) and the sample size was small. The results of the pairwise comparisons are displayed in Figures B1 and B2. Figure B1 mainly shows that there are a few dose comparison methods that perform significantly worse than the others (namely, (1%, 3 mm) gamma, SSIM and DTA), while the performance of the other methods does not significantly differ from one another. Regarding image preprocessing, only for the image normalization method for SBRT plans significant differences are present.

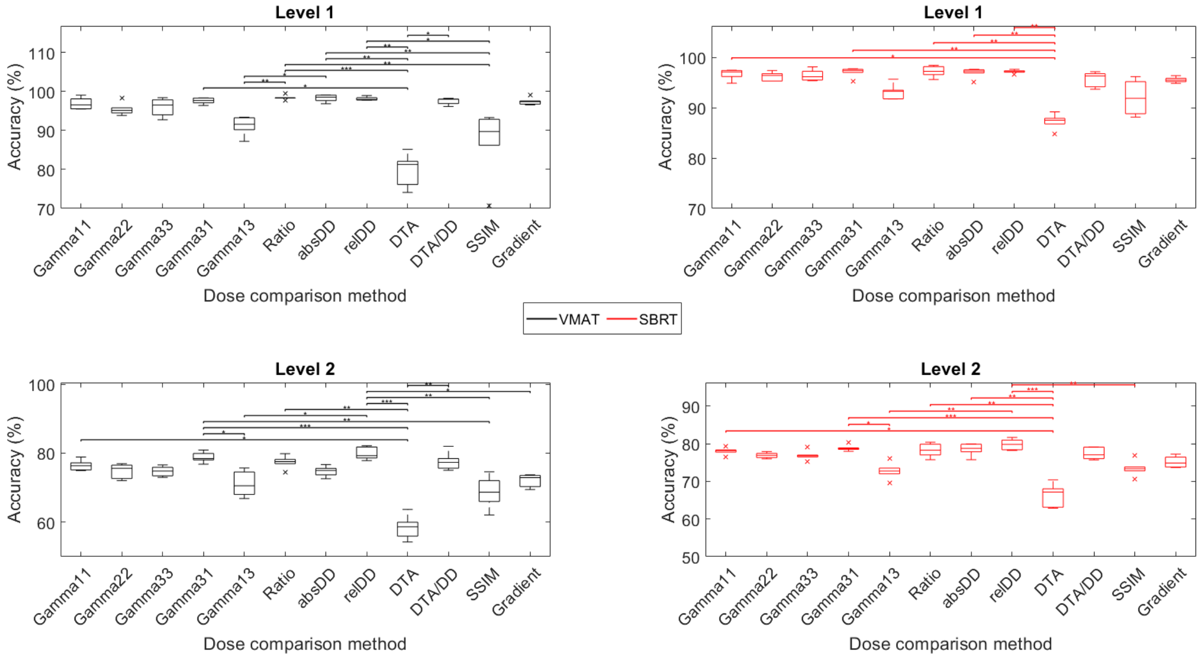

*Figure B1: Boxplots of deep learning model accuracy per dose comparison method, including statistical test results. *: p < 0.05; **: p < 0.01; ***: p < 0.001. GammaXY: (X%, Y mm) gamma map, relDD: relative dose difference, absDD: absolute dose difference, DTA: distance-to-agreement, SSIM: structural similarity index.*

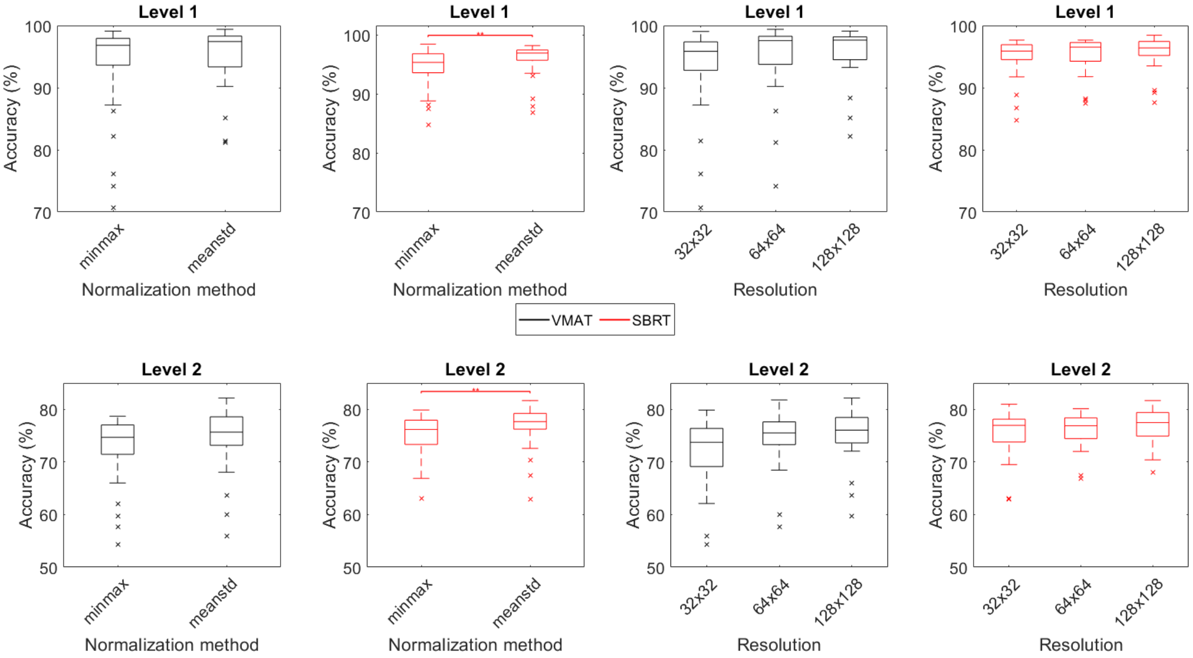

*Figure B2: Boxplots of deep learning model accuracy per image normalization method and resolution, including statistical test results. *: p < 0.05; **: p < 0.01; ***: p < 0.001.*
